# Do humanitarian missions impede the progress of pediatric cardiac surgical programs in developing and under-served nations?

**DOI:** 10.1007/s12055-024-01766-x

**Published:** 2024-06-18

**Authors:** Tom Roland Karl, Rodrigo Soto, Rob Raylman

**Affiliations:** 1 Discipline of Surgery, Paediatric Cardiac Research, University of Queensland, Brisbane, Australia; 2 Gift of Life International, Fresh Meadows, NY USA

**Keywords:** Humanitarian pediatric cardiac surgery, Humanitarian cardiac surgical team, Low-middle-income countries

## Abstract

Over the past three decades, there has been increasing interest in humanitarian pediatric cardiac surgery. While the need is undisputed, the most effective strategy remains debatable. With education, long-term sustainability, and safety as goals, we discuss the realities and possible benefits of the most frequently invoked care model, the visiting humanitarian cardiac surgical team.

## Introduction

The question posed to us by the editors of the *Indian Journal of Thoracic and Cardiovascular Surgery* is difficult to answer definitively. While it is tempting to respond with a strong “no,” the question requires a more nuanced discussion. It cannot be answered in a rigorously scientific way, as we are unable to conduct any sort of useful comparative or prospective study.

As health workers from affluent countries, we strongly support the concept that all children, of all nations, have the right to quality care for congenital and acquired heart disease. This is a premise of the World Congress of Pediatric Cardiology and Cardiac Surgery (WCPCCS), the Washington DC WCPCCS Call to Action, and various organizational charters [1]. It is a worthy goal, but not yet a reality. The authors have a cumulative experience in various aspects of humanitarian pediatric cardiac care that spans 80 countries. We believe that we (along with many other friends and colleagues working in this arena) are qualified to provide an informed commentary. We acknowledge that there may be both pragmatic and emotional components to such a thesis, and as the authors are already involved in and committed to this sort of work, there is always the possibility of “confirmation bias.” In this opinion piece, we attempt to present our concordant views.

## Discussion

Over the past three decades, there has been increasing interest in humanitarian pediatric cardiac surgery. The topic now has its own peer-reviewed literature; a PubMed search identifies well over 100 citations, the majority appearing in the last decade. Entire international meetings have been built around humanitarian cardiac initiatives, and the latter featured prominently in the 2023 WCPCCS (Global Health and Advocacy Village) [1, 2]. Most international cardiac societies have, at the least, issued strong policy statements. Databases have been established with a move to transparency in reporting outcomes in developing countries. Ethicists now debate important issues in the delivery of humanitarian care [3]. Despite all of this positive movement, governmental support in affluent countries remains elusive, and most work has been funded through non-governmental organizations (NGOs), or philanthropic and religious initiatives (additive to local resources).

In low-middle-income countries (LMICs), there has been a 50% global reduction in the mortality risk for preventable diseases over the last 30 years. On the other hand, we have not seen a corresponding decrease in the risk for non-communicable diseases, including congenital heart disease [4]. The statistics seem soberingly familiar: annually, over 1.25 million children are born with congenital heart disease, and 90% or these births occur in resourced-challenged areas [5]. Such countries tend to have higher birth rates, amplifying the burden [6]. To this, we can add the huge cohort of children with acquired rheumatic heart disease. Perhaps 90% will not have adequate access to care, translating to 200,000 *preventable* deaths per year, again, concentrated in LMICs [5, 6]. Pre-, peri-, and postoperative barriers to such access have been collated recently in an excellent review by Cheng et al. [6].

The generic term “LMIC” (used throughout this discussion) can itself be misleading. By definition, a LMIC designation implies that the gross national income per capita is in the range of 1026–3995 US dollars [7]. However, the quality and access to pediatric cardiac care may vary significantly within one LMIC, and to some extent within more affluent countries as well. For example, India and China, both listed by the World Bank as LMICs, are world leaders in pediatric cardiac surgery, with multiple centers of excellence, but a lack of universal access. In the most disadvantaged LMICs, pediatric cardiac surgery may (understandably) have a low priority. Much more basic health needs must be met in order to prevent morbidity and mortality in children without congenital heart disease. The complexity and breadth of the access issues is formidable. How can we even begin to effectively address this problem?

We (like most pediatric cardiac surgeons and cardiologists) accept that in the absence of local human and material resources, the access issue can be temporarily addressed by visiting humanitarian teams, nearly always with the long-term goal of establishing a permanent high-quality and adequately resourced local program. Failing that, indefinite support would be a second (but difficult to sustain) option. Our view (which is shared by most colleagues) is that if development of the local team is actually impeded by the presence of the visiting team, then important issues have probably been overlooked or inappropriately addressed, and some re-thinking or paradigm shift is needed [1, 8].

The question of intermittent short visits vs. long-term in situ assistance will always be controversial. There are a few organizations that have managed to employ excellent full-time pediatric cardiac surgeons who can dedicate more time to specific host institutions [9, 10]. Likewise, there are a few senior members of the specialty who have relocated more or less permanently, but they are exceptions. Consequently, most organizations rely on volunteers for short (1–2 week) intermittent visits from humanitarian teams. Who are these volunteers, and why do they do it?

The majority of pediatric cardiac surgeons working in more affluent countries have at some time been interested and/or directly involved in humanitarian surgical work. It is fair to say that as a group, we (and our professional societies) tend to be socially and politically aware, and by nature maintain a service-oriented outlook. A select few individuals (including some now-iconic pioneers) have even built their academic and clinical careers around humanitarian initiatives. Lin et al. have assessed the high interest level in global cardiac surgery among trainees in US programs [11]. Eighty-three percent expressed interest in participation, but lack of both funding and institutional support were identified as major barriers.

Humanitarian work is probably a poor choice for surgeons who have struggled significantly in their own careers at home, for whatever reason, or who lack experience with an adequate number of cases of sufficient complexity. The experience issue is not limited to cardiac surgery. Traynor et al. have suggested that Accreditation Council for Graduate Medical Education pediatric general surgical trainees will not acquire sufficient experience in orthopedics and obstetrics to meet the needs of humanitarian missions [12]. Ideally, one would want well-qualified successful team members (in all related disciplines) to serve as examples for developing teams. As a reality check, the majority of such individuals have hard-won academic appointments and work full time in university hospitals, with large caseloads, formidable research responsibilities, and administrative time commitments. Such individuals would rarely be able to relocate long-term with the expectation of retaining their jobs at home.

Although historically many cardiac surgeons have had the ability to dictate their own work and travel schedules, it is becoming more difficult to do so, to the point where time away from an institution (academic or otherwise), for any reason, can become a liability. Furthermore, most humanitarian surgery is done on a volunteer basis, with neither salary nor (in some cases) travel support, adding complexity to the equation. Although cardiac surgeons are well-paid in most parts of the world, many have incurred huge debts during their education, and have additional living and family expenses etc. Government support is almost unknown, and very few institutions (university hospitals, charities, NGOs, etc.) will be willing to sponsor humanitarian in situ cardiac teams on a long-term basis. Our point here is that creating a volunteer team willing and able to completely relocate to a foreign country for the time it takes to build and stabilize new pediatric cardiac service is an admirable and effective (but quite unlikely) prospect.

The unfortunate and somewhat pejorative terms “surgical tourism” and “surgical safari” were coined decades ago by a few surgeons who felt that brief operating trips to underserved areas were more self-serving than humanitarian in nature, and did little to promote establishment of permanent facilities or long-term educational programs. Evidence does not really support this view; in the current era, we have numerous counter-examples scattered throughout the world. There are multiple freestanding independent local teams now operating without direct assistance from foreign humanitarian groups, some at an outstanding level. Most were initially completely dependent on such assistance, and eventually gained autonomy. Others received temporary assistance at various stages in their development as a means to improve capacity and outcomes. One might say that the goal of the visiting teams in this context is their own obsolescence. It is difficult to imagine that these same home teams (despite the depth of local talent and dedication) could have done this completely on their own over the same time scale. The primary role of the visiting team then is to initiate, catalyze, and accelerate the process. One could even argue that allowing things to take an uncertain natural course has negative ethical implications.

Fenton et al. have emphasized the potential cost-effectiveness of pediatric cardiac surgery in under-served nations, acknowledging that “in the poorest countries, where access to drinking water and basic nutrition is problematic, it will often be more appropriate to focus on these issues first” [13]. While this important point may seem obvious, host team members are often keen to elevate the level of care they can deliver in their specific cardiac specialties as well. In some instances, the chance to do so is what keeps talented people from pursuing a career opportunity outside their home country. Understandably, surgeons and cardiologists in developing areas have had to seek training abroad, and are highly motivated to apply and maintain their hard-won expertise to the fullest extent at home. Visiting teams can certainly have a meaningful role in this process, both as promoters and modulators.

Cardarelli et al. have shown that the intermittent visits model has also been highly cost-effective across 10 LMICs studied, using various quality of life and financial long-term indicators [14]. Rahman et al. have analyzed data from the International Quality Improvement Collaborative, which tracks outcomes of surgery for congenital heart disease in various low-resource settings [15]. The authors noted “a moderate relationship among wealth, healthcare investment, and malnutrition.” There was, however, “significant variation, including superior results in many countries with a low gross domestic product per capita.” The authors interpreted these findings as supportive of investment in congenital heart disease procedures in low-resource settings.

Various thoughtful proposals for the best methodology for establishing a program and meeting its educational and clinical needs can be found in the cardiac surgical literature [16]. Although there are some common themes, each host center has its own set of circumstances, incentives, goals, and human/financial resources. Important issues that demand consideration include abilities of the blood bank, intensive care unit and medical sub-specialty support, and others. The patient characteristics may vary as well. Complexities of national and local politics, culture, and religion may be critically important factors. The unifying theme, if there is one, is that the scenario of needy children lacking access to quality cardiac surgical care creates a tragic situation, which we will always find difficult to ignore.

Gift of Life International (GOLI) is a well-established and highly successful humanitarian organization providing and promoting pediatric cardiac care in numerous locations throughout the world [17]. The emphasis is *sustainability*, defined as development of team capacity to safely treat children and securing of long-term government financial support. GOLI has assisted 22 programs over the past two decades, located in Africa, the Middle East, the Philippines, Central and South America, Eastern Europe, and the Caribbean. At least four of these centers have met our criteria for independence, and others are close to meeting this goal. The GOLI protocols followed for stepwise new program development are available online [18]. GOLI accepts that the onus is on NGOs to strategically address the needs of countries individually, and that no single protocol can serve all situations. The principal modus is intermittent “training visits” rather than “missions,” emphasizing the importance of balancing urgent patient care needs and program building. The initiative is therefore viewed as a “partnership” rather than a “service” per se. Specific strategies employed successfully by other humanitarian organizations are also available online and in the literature [4].

Pediatric cardiac surgery is expensive in any context. Access to supplies and equipment (especially expensive consumables), limitations of physical facilities, and funding educational needs can be major issues. Funding will probably always be problematic in resource-challenged areas. Governmental assistance for this sort of work is variable, but generally hard to come by, both in the team’s country of origin and the host country. Fortunately, donor support of NGOs at home has been generous in many affluent countries, although not completely predictable. This may be complicated by the large number of humanitarian organizations competing for the same funds, without any type of centralization or coordination of efforts. In theory, this is a modifiable issue, but action toward such a strategy has been minimal [4]. The number of active humanitarian groups worldwide probably exceeds 100, based on the number of organizations (126, from 38 countries) that were signatories to the Washington DC WCPCCS Call to Action in 2023 [1]. Host country financial support is likewise variable, and often must be continually re-negotiated. A major role of visiting teams is absorption of some of these costs and provision of supplies required for delivery of care. The long-term plan includes guidance for the host institution in negotiating with suppliers, fund raising initiatives, and interaction with charitable groups. In all of these economic initiatives, it is advantageous to have someone with expertise in business and international finance who can concentrate on the vital but sometimes difficult negotiations, as this is a role for which the majority of surgeons will find themselves unprepared.

Requests for humanitarian assistance are almost always initiated by the host institution or country. Cardiologists in developing areas are often excellent, and very experienced in diagnosis and long-term care of children with complicated congenital heart disease. At the same time, we often sense their frustration in their inability to offer definitive surgical treatment to all who need it. Surgeons being surgeons, whatever our background, experience, or venue, we always desire to take our work to the next level and improve our results. Training visiting teams can accelerate this process in a number of ways, but it must be understood that there is a large variability in host institutions seeking humanitarian assistance. Such a request is usually a policy decision, not a guarantee that all practitioners in the host institution will be co-operative or enthusiastic. The presence of a visiting team, despite the potential benefits, is an enormous imposition on the host institution, with a guarantee of interrupting the usual daily activities. Visiting team members must therefore be diplomats as well as healthcare workers. A healthy, supportive, inclusive, and mutually respectful interaction with the host team is probably the most critical aspect of any mission. If successful, a collegial and long-lasting relationship can result, with the possibility of lifelong friendships and great mutual benefit.

Training visits are often featured in local press releases, often with an emphasis on what the humanitarian team have brought, such as a particular patient story with an improbably good outcome and success with a novel operation or device. Fenton et al. have studied and emphasized the importance of giving due credit to the local team that the visiting team is (or should be) trying to empower [19]. At the same time, visiting team members must be prepared to respectfully accept protocols which do not necessarily apply in their home units, and be willing to build on what is available locally, always leading by example and proof of concept.

In some cases, there can be conflicting agendas, relating to the immediate needs of the patients actually presented to us for treatment during a visit, the present and future needs of the institution and host country, and needs of the visiting team and sponsoring organization. For example, patient safety and avoidance of morbidity and mortality are paramount for the visiting team, as a major adverse outcome can easily quell the local enthusiasm for advancing the program. At the same time, risk aversion might disqualify many needy children for their one shot at long-term survival, despite the odds. Triage and appropriate, well-informed case selection and timing, in our experience, can create major challenges for both the visiting and host teams.

By way of example, we have operated in institutions wherein the local team were enthusiastic about introducing advanced treatments such as complex neonatal repairs and cardiac transplantation, despite the fact that the ICU lacked basics such as soap and clean hand towels. We have also experienced missions during which we cancelled cardiac cases to offer our own team’s ventilators to otherwise healthy infants with acute bacterial pneumonia, who in some cases were being hand-bagged by staff and parents round the clock. In this sense, the benefits of humanitarian missions go beyond the small proportion of cardiac cases that can be treated during a 2-week visit. The introduction of technology required for an open-heart operation has huge benefits for non-cardiac patients as well, including ventilation strategies, invasive monitoring, imaging, nutritional support, novel medications, and other areas.

The concept of a decision to perform only “straightforward” cases in the humanitarian setting is likewise challenging. A newly presenting 6-year-old patient with tetralogy of Fallot (TOF) and a hematocrit above 70%, complicated by coagulopathy, malnutrition, and neurologic injury, would be quite rare in affluent countries, but not in less developed or impoverished parts of the world. Most of us would agree that care of such patients can be at least as challenging as a critically ill newborn with hypoplastic left heart syndrome encountered at home. Furthermore, visiting teams, despite their expertise in infant TOF repairs, may have little or no experience with such late presenting cases. Even less anatomically complex cases such as ventricular septal defects typically can present decision-making challenges relating to late presentation and the prospect of pulmonary vascular obstructive disease. The latter could probably be considered the index case for mission teams, who are presented with a change to radically alter a child’s life.

Language issues deserve consideration. Unlike the rest of the world, native English speakers as a group are notoriously unmotivated in acquiring second or third languages, despite the fact that communication with colleagues and families is so critical to success. Ideally, speakers of the native language(s) of the host country should be included within a humanitarian team. If this is impossible, then adequate medical translational services should somehow be assured on site at all times. This is important not only for assurance of quality care, but also to promote maximum inclusivity of the local team in the many critical informal conversations required to provide that care. These types of discussion are priceless in achieving educational goals.

Academic activity is essential for all cardiac specialties, both for self-education, credentialing, and forward movement. Another important function of visiting teams is to encourage local team members to involve themselves in research initiatives. The time to start is at the first encounter, with comprehensive prospective collection of clinical data, and identification of questions that might be addressed. Participation in international meetings, including submission of abstracts, should be encouraged and facilitated as much as practically possible. As a start, there is almost always an interesting case report to be submitted, and we try to encourage this with all host teams [20, 21]. Acceptance of a paper by a peer-reviewed journal can be a very positive team-building event for all involved. Finally, writing and submission of a paper based on clinical experience on a particular mission should *always* include local co-authors. Local team members should be encouraged and supported in involvement with international societies and their database initiatives as well. We have had success in helping local team members find time-limited training opportunities abroad.

In fairness, as mentioned, there are other models available to deliver humanitarian pediatric cardiac assistance. An argument can be made for not reinventing the wheel, when high-quality care is readily available in affluent countries [22, 23]. Evidence suggests that patients can be transported to such centers from abroad, and treated with outcomes similar to those achieved for local patients. In many regions, good ongoing care can be provided upon return. However, with such a model the patients are (by definition) highly selected. Lacking development of local facilities, the vast majority of children needing treatment still cannot receive it. The global patient and family benefit is therefore limited in comparison to what might be achieved over the same interval using the same funding to promote development of a local program. This is not meant to diminish the huge benefits that have been realized by the selected patients and their families, which are of course life-changing. We note that in the USA and some other affluent countries that it is currently nearly impossible to organize charitable or meaningfully discounted care for children brought from overseas. In general, some of the issues raised herein reinforce the difficulty in moving completely away from the visiting team or NGO humanitarian mission model [24].

Humanitarian work in pediatric cardiac surgery is an excellent opportunity to address some of the inequities in global child health. It is easy to decry the tragic situations that congenital heart disease inflicts on society, but far more meaningful and rewarding to step up to offer one’s expertise to the many local teams and patients that can benefit. This must be combined with meaningful and ongoing policy activism at the national and global level [1]. There is clearly much more to be said on the topic, but as well-stated by Corno, “Success should not be measured by the number of successful operations on any given mission, but by the successful operations that our colleagues perform after we leave” (25).

## Conclusion

Our key points may be summarized as follows:

1. The current level of access to care for children with congenital heart disease is, in many parts of the world, unacceptable.
2. Visiting teams from affluent countries can immediately begin to address this problem.
3. The long-term goals should always include establishment of an independent and well-funded team delivering comprehensive high-quality pediatric cardiac care.
4. While the training visit model may be imperfect, we have ample proof of concept that it is effective in important ways, and does promote growth and development that would otherwise require a much longer time scale.
5. For many host countries, this model is also the only feasible solution for meeting urgent local needs.
6. We find little (if any) evidence to support the concept that visiting humanitarian teams providing training missions to LMICs impede development of the local team.

## Declarations

### Disclaimer

All authors are involved (clinically and/or administratively) with Gift of Life International.
